# Parecoxib sodium alleviates ischemia reperfusion‐induced pulmonary injury via inhibiting ERK/NF‐κB and further activating the HIF‐1α pathway

**DOI:** 10.1002/iid3.684

**Published:** 2022-08-17

**Authors:** Jiantao Guo, Yiping Yang

**Affiliations:** ^1^ Department of Anesthesiology, Taizhou First People's Hospital Huangyan Hospital Affiliated to Wenzhou Medical University Taizhou Zhejiang China

**Keywords:** hypoxia‐inducible factor‐1α, inflammatory response, ischemia reperfusion, oxidative stress, parecoxib

## Abstract

**Introduction:**

The lungs are extremely vulnerable to ischemia/reperfusion (I/R), which is characterized by intense inflammation, oxidative stress, alveolar damage, and vascular permeability. Parecoxib sodium (Pare) has been shown to exert protective effects against multiple I/R‐induced tissue injuries. However, its role in I/R‐induced lung injury remains unknown. This study aimed to reveal the roles and mechanisms of Pare in pulmonary I/R injury.

**Methods:**

Sixty‐six rats were randomly divided into three groups: The sham‐operated group, the pulmonary I/R group, and the Pare‐pretreated I/R group. Pare at 10 mg/kg or saline (vehicle control) were intraperitoneally administered to rats once per day for 5 consecutive days before ischemia. Serum and tissue samples were harvested following 2 h of reperfusion. The oxygenation index (OI) and alveolar‐arterial oxygen partial pressure difference (PA‐aO_2_) were analyzed. The levels or activities of malondialdehyde, superoxidase dismutase, catalase, glutathione peroxidase, intracellular reactive oxygen species, tumor necrosis factor‐α, interleukin (IL)‐6, and IL‐8 were examined. The mitochondrial membrane potential was measured. The protein expression levels of the extracellular signal‐regulated kinase (ERK), nuclear factor‐κB (NF‐κB) and their phosphorylated forms, and hypoxia‐inducible factor‐1α (HIF‐1α) were detected. Histological changes were observed using hematoxylin and eosin staining. Moreover, the survival rate following pulmonary I/R injury was recorded daily.

**Results:**

Pare significantly increased the OI, decreased the PA‐aO_2_, increased the levels of antioxidants, while decreasing the levels of oxidants, and alleviated mitochondrial dysfunction and the histopathological damage induced by I/R. Furthermore, Pare inhibited the expression of proinflammatory cytokines, suppressed the activation of ERK and NF‐κB, further increased HIF‐1α expression, and significantly improved the rat survival rate.

**Conclusions:**

Pare pretreatment attenuated lung I/R injury by inhibiting oxidative stress and the inflammatory response possibly via inhibiting the activation of the ERK/NF‐κB pathway and further activating the HIF‐1α pathway.

## Graphical abstract

Parecoxib sodium alleviates ischemia/reperfusion (I/R)‐induced pulmonary injury via inhibiting extracellular signal‐regulated kinase/nuclear factor‐κB and further activating the hypoxia‐inducible factor‐1α pathway

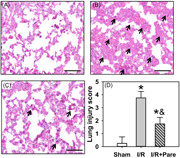

## 1 INTRODUCTION

The occurrence of ischemia and subsequent reperfusion (I/R) is unavoidable during organ transplantation. I/R is characterized by cell damage in certain organs under hypoxic conditions, followed by the sudden restoration of oxygenation to tissues,^1^ which can cause devastating consequences in transplant recipients. Severe primary graft dysfunction has an incidence of 30% within 3 days of transplantation, and it also leads to graft rejection and mortality in recipients at 1‐year posttransplantation.^2^ Lung I/R injury is a major contributor to pulmonary graft failure and the high mortality rate following lung transplants. Lung I/R injury can disrupt the permeability barrier of alveolar capillaries, promote the exudation of plasma components, cause interstitial edema and bleeding, and may finally result in ventilation dysfunction.^3^ Therefore, strategies for the prevention of I/R injury are urgently required to ameliorate both the short‐ and long‐term outcomes following lung transplantation.

The pathogenesis of pulmonary I/R is complex and involves various pathways and pathophysiological processes, including oxidative stress, inflammatory responses, endoplasmic reticulum stress, calcium overload, apoptosis, and autophagy.^3^, ^4^ Ischemia significantly increases calcium levels both inside cells and in the mitochondria by destroying adenosine triphosphate enzyme (ATPase)‐dependent ion transport, and decreasing intracellular ATP and pH levels.^5^ The ATP shortage impairs the cell volume and causes the lysis of organelles and plasma membranes.^5^ Subsequent reperfusion accelerates reactive oxygen species (ROS) production, proinflammatory immunocyte aggregation and endoplasmic reticulum stress, which further exacerbate tissue injury.^6^, ^7^ These changes eventually cause the opening of mitochondrial permeability transition pores and aggravate I/R‐induced cell lysis and death.

Cyclooxygenase (COX) is a key rate‐limiting enzyme involved in prostaglandin synthesis. COX‐2 is a COX isozyme and is predominantly located at the nuclear membrane; thus, released prostaglandins would preferentially enter the nucleus and regulate gene transcription.^8^ The expression of COX‐2 post‐I/R has been found to increase, while the inhibition of the COX‐2 levels has been found to reduce inflammatory responses and alleviate I/R‐induced injury.^9^ Parecoxib sodium (Pare), a nonsteroidal anti‐inflammatory drug commonly used for postoperative pain, is a selective inhibitor of COX‐2.^10^ Numerous studies have demonstrated that Pare plays a protective role in various organs, including the brain,^11^ heart,^12^ liver,^13^ and kidneys^14^ in I/R animal models. Such a protective role of Pare is mainly mediated via anti‐inflammation, anti‐oxidation, as well as the regulation of apoptosis,^11^, ^12^, ^13^, ^14^ highlighting its superiority and potential value in clinical application. It has been reported that Pare can prevent I/R injury in a dose‐dependent manner, possibly by suppressing the inflammatory reaction and apoptosis of intestinal mucosal epithelial cells.^15^ However, whether Pare also exerts the same protective effects against pulmonary I/R and the underlying mechanisms involved have rarely been reported. In the present study, a rat model of pulmonary I/R was established using the noninvasive vessel clamping method to assess the effects of Pare on I/R injury. In addition, mechanisms related to oxidative stress, inflammation, and changes in the extracellular signal‐regulated kinase (ERK)/nuclear factor‐κB (NF‐κB), hypoxia‐inducible factor‐1α (HIF‐1α) pathways were explored.

## 2 MATERIALS AND METHODS

### 2.1 Animals

A total of 66 adult male Sprague–Dawley rats (specific pathogen free‐grade, 202006‐dw‐0251) at 8 weeks of age (weighing ∼250 g) were commercially purchased from Hangzhou Yingyang Biomedical R&D Center (SYXK 2020‐0024). The rats were individually kept in standard cages placed in a room (temperature, 25°C; relative humidity, ∼50%, 12 h light/12 h dark cycle). All experimental procedures on the rats were reviewed and approved by the Experimental Animal Ethics Committee of Hangzhou Yingyang Biomedical R&D Center (approval no. EYOUNG‐20210304‐01), and performed strictly following the Guide for the Care and Use of Laboratory Animals by the National Institutes of Health (revised version in 1996). Ultimately, the rats received euthanasia via CO_2_ asphyxiation at a flow rate of 30% chamber vol/min.

### 2.2 Grouping and treatment

The 66 rats were randomly divided into the following groups: The sham‐operated group (sham, *n* = 22), the pulmonary I/R group (I/R, *n* = 22), and the Pare‐pretreated I/R group (I/R + Pare, *n* = 22). Pare (cat. No. H20193247; Emeishan Tonghui Pharmaceutical Co., Ltd.) was dissolved in 0.9% saline solution and then intraperitoneally injected into the rats (at 10 mg/kg) once daily for 5 consecutive days before ischemia. The dose of Pare was determined according to several recently published studies.^12^, ^13^, ^15^ The sham and I/R groups received the same volume of 0.9% saline solution. An animal model of I/R‐induced lung injury was established according to previously published studies.^16^, ^17^ On the day of the procedure (namely, Day 0), rats were intraperitoneally injected with pentobarbital (40 mg/kg body weight) to induce the anesthesia and ventilated by tracheostomy. Each rat received an intramuscular injection of 0.2 mg atropine and intravenous injection of 50 U heparin before the experiment. The left lung was mobilized through an anterolateral thoracotomy and the left pulmonary artery, veins, and bronchus were occluded using a noninvasive microvascular clamp to induce the ischemia. At the end of the 1‐h ischemic period, the clamp was removed and the lung was ventilated and reperfused for 2 h. The anesthesia was maintained for all 3 h during the I/R procedure with inhaled halothane. Blood and pulmonary tissues were then collected and used for subsequent assays. Rats after blood/tissue collection received euthanasia via CO_2_ asphyxiation. The survival of the rats was recorded daily for 7 consecutive days post‐I/R injury (namely, from Day 1 to 7). Ten independent rats in each treatment group were used. The humane endpoint was set as the following: body temperature decreased by 4–6°C, body weight decreased by more than 10%, showing lethargy, low alertness, rough appearance, hunched posture, indicating illness, pain, or distress. The health and behavior of rats were monitored twice daily.

### 2.3 Blood gas analysis

Following 2‐h of reperfusion, blood samples were obtained from the femoral artery. Several parameters for lung ventilation function, such as partial pressure of oxygen (PaO_2_) and partial pressure of carbon dioxide (PaCO_2_) were measured and recorded using a blood gas analyzer. The alveolar‐arterial oxygen partial pressure difference (PA‐aO_2_) = (atmospheric pressure − saturated water vapor pressure) × fraction of inspired oxygen (FiO_2_) − PaCO_2_/0.8 − PaO_2_. The oxygenation index (OI) = PaO_2_/FiO_2_. A total of four independent rats from each group were used.

### 2.4 Biochemical assays

Tissue samples were quick‐frozen in liquid nitrogen and then homogenized with 4°C cold PBS. The homogenized samples were then centrifuged at the speed of 4000*g* at 4°C for 20 min. The supernatants were harvested to detect the malondialdehyde (MDA) level using an MDA Assay Kit (cat. no. S0131S; Beyotime Biotechnology). MDA is the final product resulting from lipid breakdown and oxidative stress, and it is considered a good indicator of free radical‐induced lipid peroxidation. MDA can react with thiobarbituric acid (TBA) to form a red product MDA‐TBA with a maximum absorption at 535 nm, thus the content of MDA can be detected by chromatometry. Superoxidase dismutase (SOD) activity in pulmonary tissues was measured using a microplate reader at the absorbance of 560 nm according to the xanthine oxidase method, with a SOD assay kit (cat. no. S0109; Beyotime Biotechnology). The kit for catalase (CAT) activity was purchased from Nanjing Jiancheng Bioengineering Institute (cat. no. A007‐1‐1) and the absorbance was measured at 405 nm. The kit for determining glutathione peroxidase (GSH‐Px) activity was purchased from Nanjing Jiancheng Bioengineering Institute (cat. no. A005‐1‐1) and the absorbance was measured at 412 nm. In total, four independent rats from each group were used.

### 2.5 Isolation of primary type II alveolar epithelial cells

Rats after the 3‐h I/R treatment were fixed and received tracheotomy and intubation. After the thoracic cavity was exposed, pulmonary artery lavage was conducted with 50 ml phosphate‐buffered saline (PBS) to clean the vascular bed. Bilateral lungs were then collected and placed in the 10 cm culture dishes. Alveolar lavage was conducted with 10 ml PBS and repeated three times, followed by digestion in trypsin at 37°C. Ten minutes later, tissues were cut into small pieces (∼1 mm^3^ each) and digested in 0.025% DNase, which was then terminated by adding fresh Dulbecco's modiﬁed Eagle's medium (DMEM). The mixture was gently shaken in the water bath at 37°C for 5 min and successively filtered through 150, 20, and 10 μm sieves. Finally, the suspension was centrifuged at 4°C for 10 min. The precipitant was collected and suspended in DMEM.

### 2.6 Intracellular ROS detection

The intracellular ROS levels were detected using a commercial ROS Assay Kit (cat. no. S0033S; Beyotime Biotechnology) according to the manufacturer's instructions. Briefly, the primary type II alveolar epithelial cells following I/R were rapidly isolated and seeded in 96‐well plates in triplicate in each group. The cells were then incubated with 10 μM 2′,7′‐dichlorofluorescein diacetate (DCFH‐DA) for 30 min at 37°C in the dark, followed by rinsing with PBS three times to remove the excess DCFH‐DA. Finally, fluorescent signals were detected using a fluorescence spectrophotometer at an excitation wavelength of 488 nm and an emission wavelength of 525 nm (BioTek Instruments Inc.). DCFH‐DA can be easily oxidized into fluorescent dichlorofluorescein by intracellular ROS and is thus used to quantify the ROS level. In total, three to four independent experiments were performed and four independent rats from each group were used.

### 2.7 Measurement of mitochondrial membrane potential

Mitochondrial membrane potential (MMP) was detected using an Enhanced MMP Assay Kit with JC‐1 (cat. no. C2003S; Beyotime Biotechnology) according to the manufacturer's instructions. JC‐1 is an ideal fluorescent probe widely used for MMP detection. Under normal conditions, JC‐1 accumulates in the mitochondrial matrix and agglomerates into J‐aggregates that emit red fluorescence. When MMP decreases, J‐aggregates decompose into a monomer form that emits green fluorescence. Therefore, the percentage of depolarized cells can be measured by the statistical analysis of different fluorescence signals. Briefly, the primary type II alveolar epithelial cells following I/R were rapidly isolated and seeded in 96‐well plates in triplicate in each group. The cells were then incubated with JC‐1 at 37°C for 20 min. Finally, the samples were tested using a flow cytometer (BD Biosciences). In total, four independent rats from each group were used.

### 2.8 Enzyme‐linked immunosorbent assay

Kits for tumor necrosis factor (TNF)‐α (cat. no. PT516; Beyotime Biotechnology), interleukin (IL)‐6 (cat. no. PI328; Beyotime Biotechnology), and IL‐8 (cat. no. RA20553; Bioswamp) were used to measure the levels of proinflammatory cytokines in serum. The absorbance was measured at a wavelength of 450 nm using a microplate reader (Model 550; Bio‐Rad Laboratories Inc.). In total, four independent rats from each group were used.

### 2.9 Western blot analysis

Lung samples were quick‐frozen in liquid nitrogen and then homogenized in a cold radioimmunoprecipitation assay buffer containing the cocktails of protease and phosphatase inhibitors (cat. no. PPC2020; Sigma‐Aldrich). These samples were centrifuged at a speed of 12,000*g* at 4°C for 45 min, and lysates were collected. A Bradford assay was then performed to determine the protein concentrations. A total of 30 μg proteins in each sample were boiled at 100°C, loaded onto 10% sodium dodecyl sulfate (SDS) gel, subjected to SDS‐polyacrylamide gel electrophoresis, and then transferred to polyvinylidene difluoride membranes (cat. no. IPVH00010; Millipore). The blots were subsequently blocked in 5% nonfat milk at room temperature for 1 h, washed three times in PBST (15 min each time), and then incubated with specific primary antibodies at appropriate dilutions at 4°C overnight. On the following day, the blots were again washed three times in PBST and incubated with specific anti‐mouse or anti‐rabbit secondary antibodies at room temperature for 1 h. An ECL Detection Kit (cat. no. 32209; Thermo Fisher Scientific Inc.) was used to develop the protein bands. Images were captured using a MultiImage Light Cabinet (Alpha Innotech Corp.). In total, four independent rats from each group were used.

### 2.10 Histological staining

A histopathological assessment was conducted to confirm I/R‐imposed pulmonary injury and to evaluate the protective effects of Pare. The right middle lobe was collected and then immediately immersed into 4% paraformaldehyde (cat. no. P0099; Beyotime Biotechnology) for 24 h at room temperature. The next day, samples were embedded in paraffin wax and then cut into 5 μm thickness using a freezing microtome, followed by hematoxylin and eosin staining (cat. no. C0105S; Beyotime Biotechnology) to examine the histological and morphological changes. In total, four independent rats from each group were used. The histological assessment was evaluated by an experienced and blinded individual according to the standard for lung injury severity scoring with minor modification.^18^ Briefly, the scoring system of pulmonary tissue damage includes 0. normal tissue without injury; 1. minimal inflammatory change with no obvious damage to the lung architecture; 2. thickening of the alveolar septae; 3. formation of nodules or areas of pneumonitis that distorted the normal architecture; and 4. total obliteration of the field.^18^

### 2.11 Immunohistochemistry

Paraffin‐embedded lung tissue sections at 5 μm were deparaffinized in xylene and then rehydrated in a graded series of ethanol. Briefly, slides were heated at 95°C for 30 min in the citric acid buffer. After cooling, slides were incubated with 3% H_2_O_2_ for 5 min in methanol and blocked in Tris‐buffered saline containing 10% normal serum. The anti‐TNF‐α antibody (cat. no. AF8208; Beyotime Biotechnology) was applied and incubated at 1:50 dilution at 4°C overnight. The detection was conducted using a SABC‐HRP Immunohistochemistry (IHC) Kit (cat. no. P0603; Beyotime Biotechnology).

### 2.12 Statistical analysis

Data are expressed as the mean ± standard deviation. The results were statistically analyzed using the SPSS statistical software version 19.0 (SPSS Inc.). A one‐way analysis of variance followed by the least significant difference post hoc test was performed to evaluate the differences among groups. The Kaplan–Meier method with a logrank test was utilized to calculate the survival rate of the rats. *p* < .05 was considered to indicate a statistically significant difference.

## 3 RESULTS

### 3.1 Preadministration of Pare improves pulmonary ventilation function in rats with I/R‐induced lung injury

**Figure 1:**
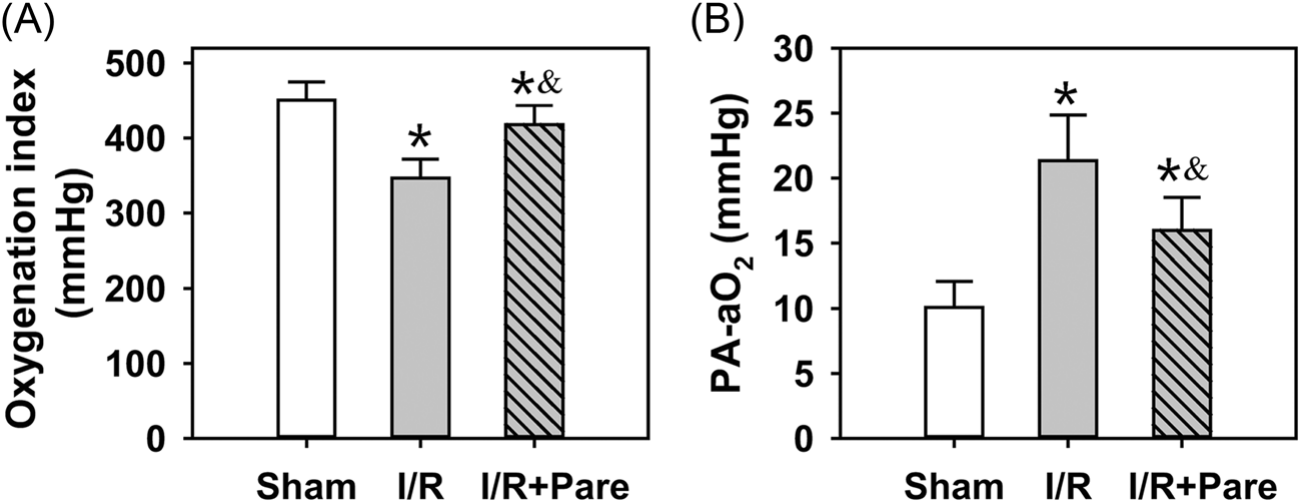
Blood gas analysis inpulmonary I/R rats. (A) Comparison of OI in three groups. (B) Comparison of PA‐aO_2_ in three groups. Bars represent the means ± SD. Data were obtained and analyzed from four independent rats in each group.^*^ *p* < .05, compared to the sham group; ^&^ *p* < .05, compared to the I/R group. I/R, ischemia/reperfusion; OI, oxygenation index; Pare, Parecoxib; PA‐aO_2_, alveolar‐arterial oxygen partial pressure difference.

OI and PA‐aO_2_ are two main indicators used to evaluate the ventilation function of the lungs.^19^ Following I/R, the blood gas analysis revealed that values of OI in the two I/R groups (I/R alone and I/R + Pare) were markedly lower than those in the sham group, while Pare significantly increased the OI compared with the I/R group (Figure 1A, *n* = 4, *p* < .05). The values of PA‐aO_2_ in the two I/R groups were markedly higher than those in the sham group, while Pare significantly decreased PA‐aO_2_ compared with the I/R group (Figure 1B, *n* = 4, *p* < .05).

### 3.2 Pare preadministration attenuates oxidative stress in rats with pulmonary I/R injury

**Figure 2:**
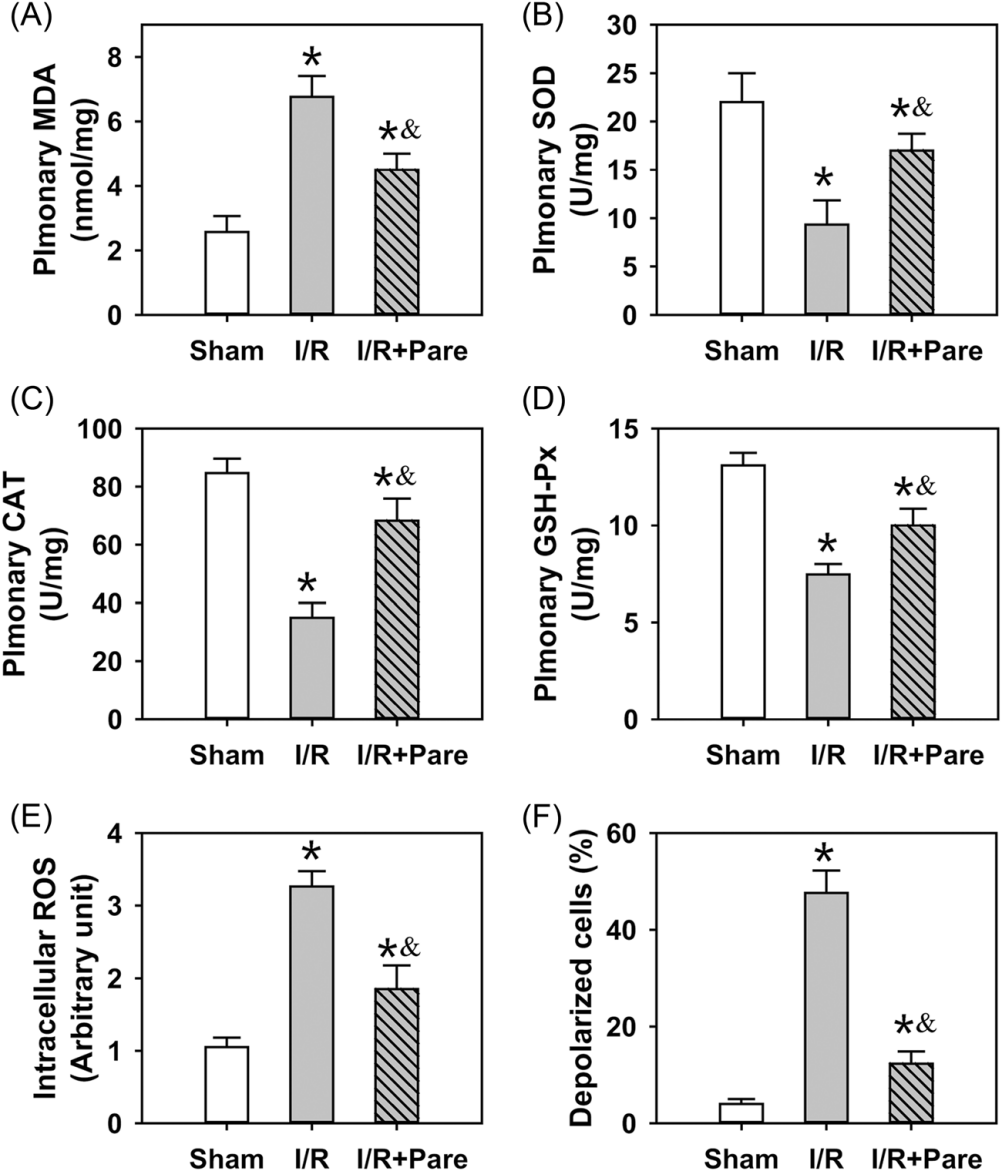
Assessment of the oxidative stress in pulmonary I/R‐induced injury. (A) Malondialdehyde (MDA) content in the lung samples of rats. (B) Superoxidase dismutase (SOD) activity in the lung samples of rats. (C) The activity of catalase (CAT) in the lung samples of rats. (D) The activity of glutathione peroxidase (GSH‐Px) in the lung samples of rats. (E)The intracellular ROS levels by a fluorescence spectrophotometer. (F) The number of depolarized cells by a flow cytometer using a JC‐1 probe. Bars represent the means ± SD. Data were obtained and analyzed from four independent rats in each group. ^*^ *p* < .05, compared to the sham group. ^&^ *p* < .05, compared to the I/R group. I/R, ischemia/reperfusion; Pare, Parecoxib; ROS, reactive oxygen species.

To assess the role of Pare in oxidative stress, the MDA level, and SOD, CAT, and GSH‐Px activities were detected in the rat pulmonary tissues. As shown in Figure 2A (*n* = 4), I/R markedly elevated the level of MDA in the lungs, a degradation product during lipid peroxidation, by ∼2.5‐fold (*p* < .05). However, Pare's pretreatment markedly decreased the I/R‐induced MDA level by 30% (*p* < .05). In addition, I/R notably decreased the activities of SOD, CAT, and GSH‐Px by 65%, 60%, and 48%, respectively, which were considerably recovered by Pare preadministration (Figure 2B–D, *n* = 4, *p* < .05). In addition, intracellular ROS production was measured. As shown in Figure 2E (*n* = 4), ROS production exhibited a significant surge  ∼threefold following I/R, while Pare pretreatment effectively suppressed this induction (*p* < .05). ROS generation is known to be closely related to the change of MMP, which is a potent indicator of mitochondrial dysfunction.^20^ Thus, the present study measured the MMP level using the JC‐1 method. As shown in Figure 2F (*n* = 4), the number of depolarized cells markedly increased following I/R injury (*p* < .05), indicating a disrupted mitochondrial function; however, Pare pretreatment significantly attenuated the I/R‐induced destructive effect on the mitochondria (*p* < .05).

### 3.3 Pare preadministration decreases the inflammatory responses in the serum of rats with pulmonary I/R injury

**Figure 3:**
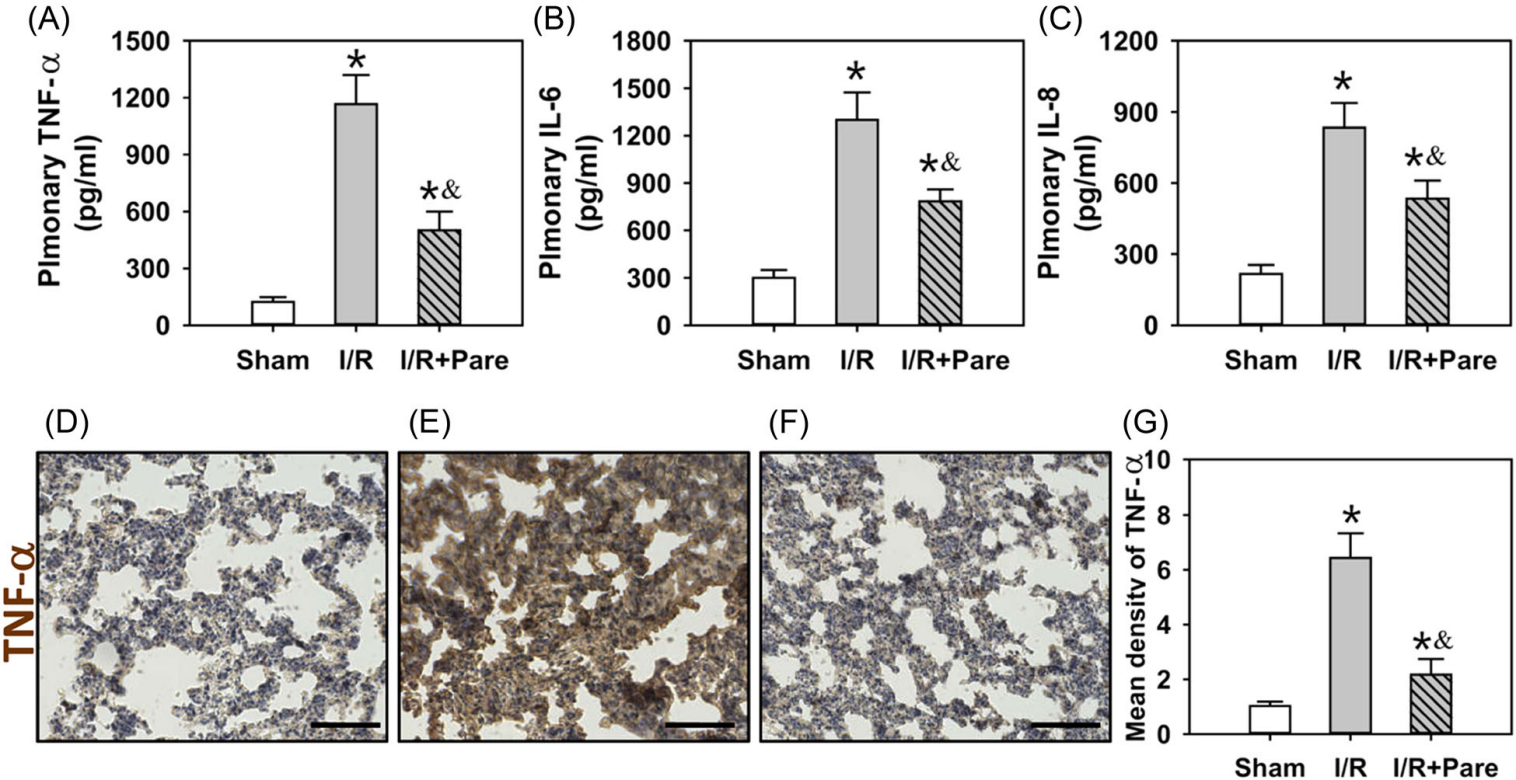
Assessment of the inflammatory responses in pulmonary I/R‐induced injury. ELISA was performed to measure the serum levels of TNF‐α (A), IL‐6 (B), and IL‐8 (C) in three groups. IHC staining of TNF‐α was shown in the sham (D), I/R (E), and I/R + Pare (F) groups. The quantitative analysis of IHC results has been generated in (G). The bars represent means ± SD. Data were obtained and analyzed from four independent rats in each group. ^*^ *p* < .05, compared to the sham group; ^&^ *p* < .05, compared to the I/R group. Scar bar = 100 μm. ELISA,enzyme‐linked immunosorbent assay;IHC, immunohistochemistry;IL‐6, interleukin 6; I/R, ischemia/reperfusion; Pare, Parecoxib; TNF‐α, tumor necrosis factor α.

To assess the potential role of Pare in inflammatory responses, the serum contents of several proinflammatory cytokines, such as TNF‐α (Figure 3A), IL‐6 (Figure 3B), and IL‐8 (Figure 3C) were measured. The biochemical results obtained using an enzyme‐linked immunosorbent assay revealed that the serum contents of TNF‐α, IL‐6, and IL‐8 were markedly elevated threefold–sevenfold post‐I/R (*p* < .05); however, Pare preadministration considerably decreased the serum contents of TNF‐α, IL‐6, and IL‐8 by 35%–55% (Figure 3A–C, *n* = 4, *p* < .05). TNF‐α was then specifically chosen for IHC staining as it is a key initiating factor in pulmonary I/R injury. Results revealed that the brown TNF‐α precipitates were significantly increased in the I/R group (Figure 3E,G) compared with the sham group which exhibited mild TNF‐α staining (Figure 3D,G). However, Pare pretreatment effectively attenuated TNF‐α precipitation induced by I/R (Figure 3F,G).

### 3.4 Pare preadministration suppresses ERK/NF‐κB and activates HIF‐1α signaling in pulmonary I/R injury

**Figure 4:**
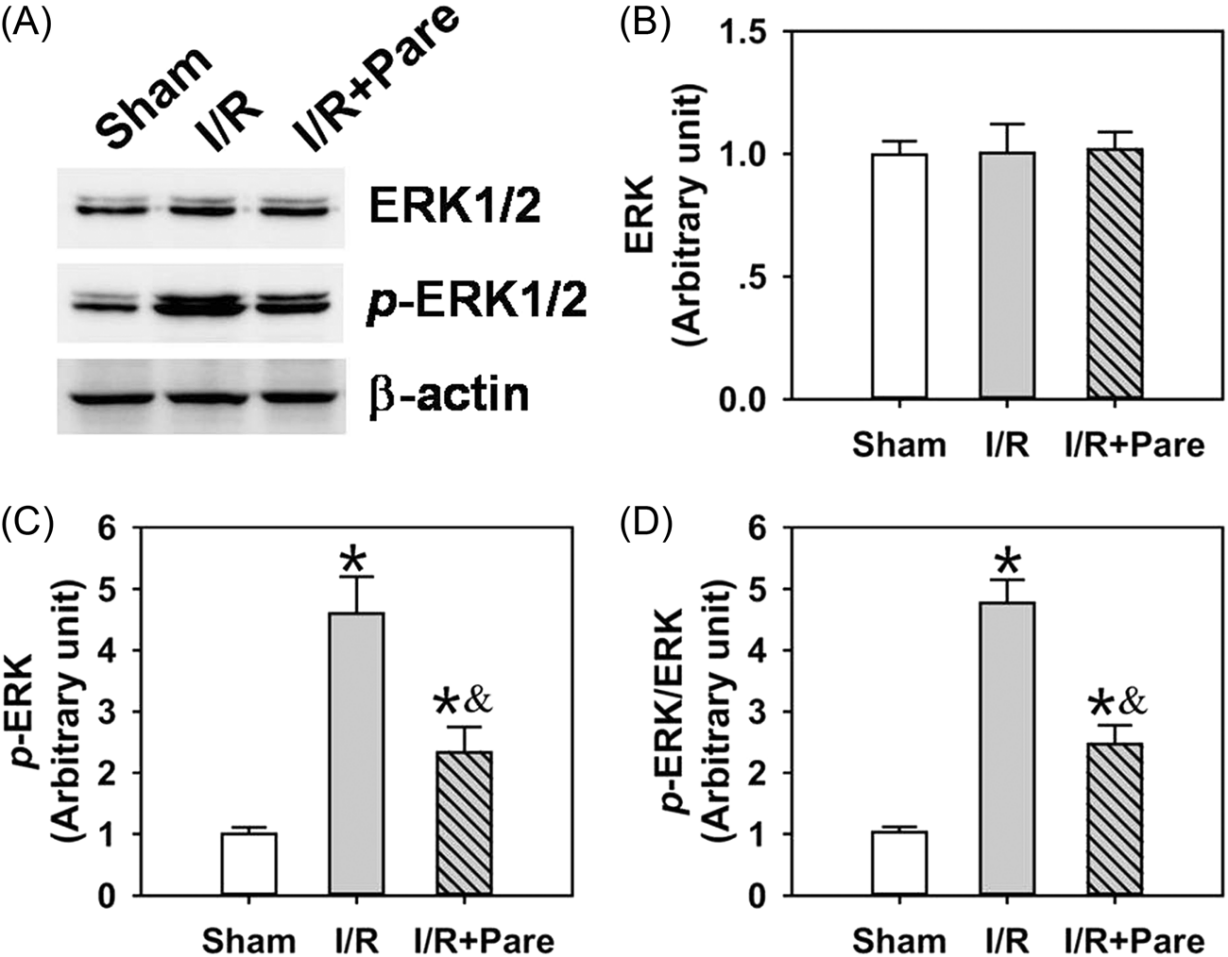
The deactivation of ERK in Pare‐mediated protection against pulmonary I/R injury. (A) Total ERK protein level and phosphorylated ERK level in rat pulmonary tissue in three groups were measured by Immunoblotting. (B, C) The densitometric analysis of the immunoblots for ERK (B) and p‐ERK (C). (D) The ratio of p‐ERK versus ERK. Each bar is the mean ± SD. Data were obtained and analyzed from four independent rats in each group. ^*^ *p* < .05, compared to the sham group; ^&^ *p* < .05, compared to the I/R group. ERK, extracellular signal‐regulated kinase; I/R, ischemia/reperfusion; Pare, Parecoxib; p‐ERK, phospho‐ERK.

**Figure 5:**
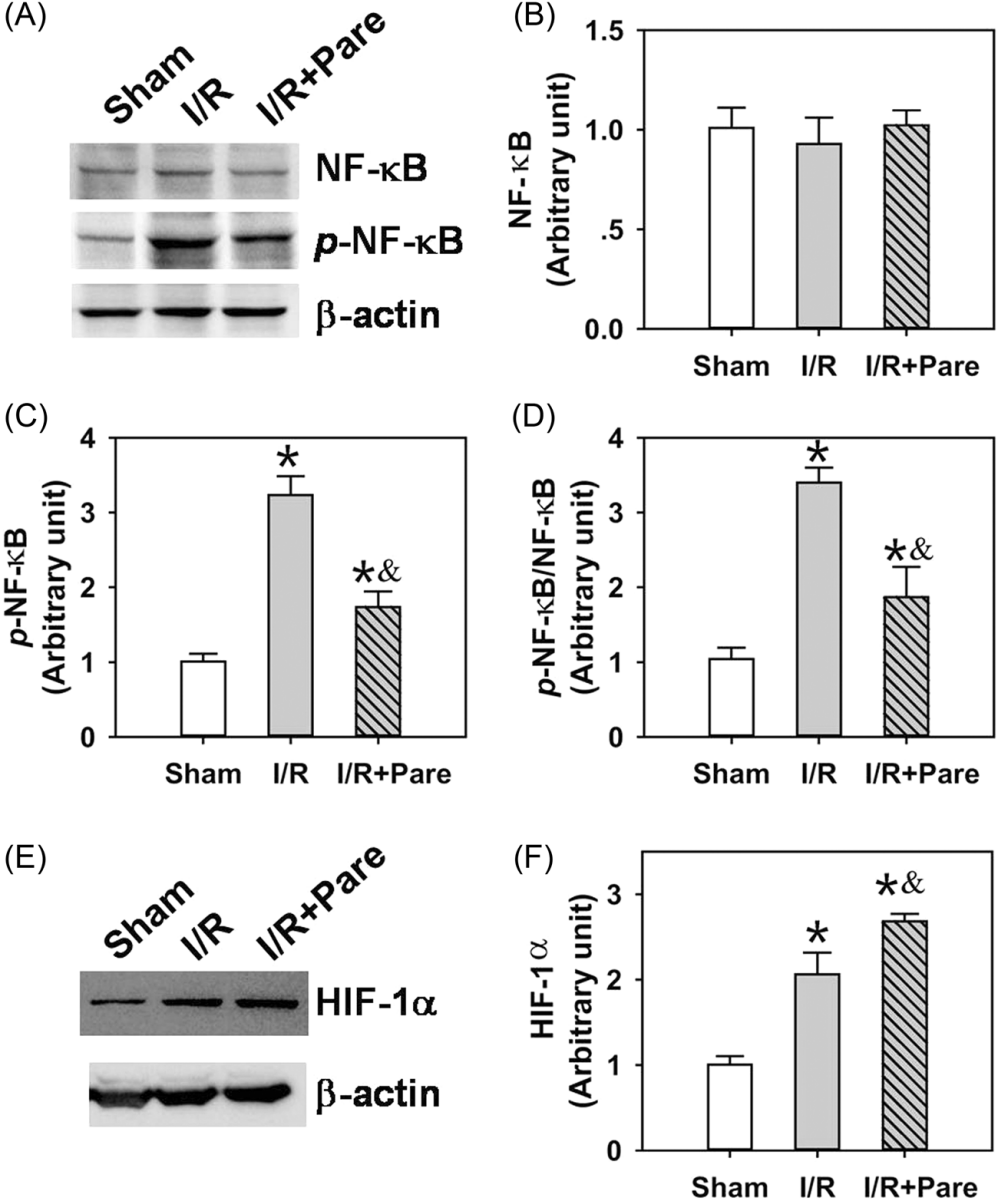
The deactivation of NF‐κB and the induction of HIF‐1α in Pare‐mediated protection against pulmonary I/R injury. (A) Total NF‐κB protein level and phosphorylated NF‐κB level in rat pulmonary tissue in three groups were measured by Immunoblotting. (B, C) The densitometric analysis of the immunoblots for NF‐κB (B) and p‐NF‐κB (C). (D) The ratio of p‐NF‐κB versus NF‐κB. (E) The immunoblot of HIF‐1α. (F) The densitometric analysis of the immunoblot for HIF‐1α. Each bar is the mean ± SD. Data were obtained and analyzed from four independent rats in each group. ^*^ *p* < .05, compared to the sham group; ^&^ *p* < .05, compared to the I/R group. HIF‐1α, hypoxia‐inducible factor‐1α; I/R, ischemia/reperfusion; NF‐κB,nuclear factor‐κB; Pare, Parecoxib; p‐NF‐κB, phospho‐NF‐κB.

The present study then investigated the underlying mechanisms through which Pare inhibited oxidative stress and inflammation in I/R‐induced pulmonary damage. ERK/NF‐κB and HIF‐1α signaling have been shown to be involved in I/R‐induced organ damage.^21^, ^22^ However, whether they are also involved in the Pare‐mediated protective effects against pulmonary I/R injury remains to be elucidated. The present study thus analyzed the changes in the protein levels of ERK and NF‐κB, as well as their corresponding phosphorylated forms phospho‐ERK (p‐ERK) and phospho‐NF‐κB (p‐NF‐κB), to reveal the inhibitory status of ERK/NF‐κB signaling. The results of western blot analysis revealed that although the total protein expression of ERK (Figure 4A,B, *n* = 4) and NF‐κB (Figure 5A,B, *n* = 4) were not affected in the two I/R groups, I/R significantly activated the expression of p‐ERK (Figure 4A,C, *n* = 4, *p* < .05) and p‐NF‐κB (Figure 5A,C, *n* = 4, *p* < .05) by threefold–fivefold. The preadministration of Pare significantly inhibited the phosphorylation of ERK (Figures 4A,C, *n* = 4, *p* < .05) and NF‐κB (Figure 5A,C, *n* = 4, *p* < .05) by ∼50%. It is important to note that the p‐ERK/ERK ratio (Figure 4D, *n* = 4) and p‐NF‐κB/NF‐κB ratio (Figure 5D, *n* = 4) were also notably decreased by Pare preadministration (*p* < .05), indicating the suppression in the ERK/NF‐κB pathway. In addition, it was also found that HIF‐α expression was markedly increased by ∼twofold following I/R, while Pare pretreatment further promoted this induction (Figure 5E,F, *n* = 4, *p* < .05), indicating the involvement of HIF‐1α‐mediated signaling.

### 3.5 Pare preadministration protects the lungs against I/R‐induced tissue injury

**Figure 6:**
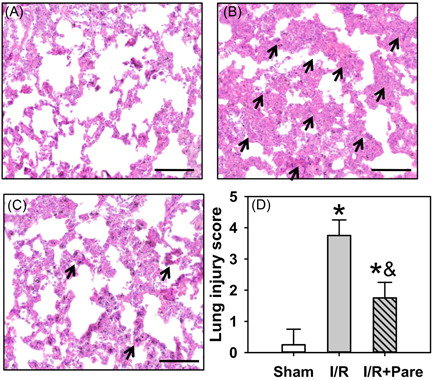
Assessment by H&E staining to confirm the effect of Pare preadministration on I/R‐caused lung damage. (A) The histological characteristics of the normal rat lung in the Sham group. (B) The histological changes of the rat lung after I/R insults. (C) The histological changes of the rat lung in Pare‐pretreated I/R group. (D) The statistic analysis of the lung damage. The scoring system of lung damage ranged from 0 (no injury) to 4 (severe injury). Black arrows indicated the site of lung injury. Each bar is the mean ± SD. Data were obtained and analyzed from four independent rats in each group. ^*^ *p* < .05, compared to the sham group; ^&^ *p* < .05, compared to the I/R group. Scar bar = 100 μm. H&E, hematoxylin and eosin; I/R, ischemia/reperfusion; Pare, Parecoxib.

The histological images revealed that under normal conditions the alveolar cell structure was clear and intact, and the alveoli did not exhibit obvious exudation (Figure 6A,D, *n* = 4). However, the I/R insult significantly disrupted the alveolar structure, promoted the interstitial edema and the leakage of erythrocytes from the alveolar cavity, accompanied by the extensive infiltration of inflammatory cells into the alveolar wall (black arrows, Figure 6B,D, *n* = 4, *p* < .05). However, pretreatment with Pare before the I/R insult significantly protected the alveolar structure, improved the condition of interstitial edema and erythrocyte leakage, and ameliorated the inflammatory cells infiltrating in the alveolar wall (black arrows, Figure 6C,D, *n* = 4, *p* < .05).

### 3.6 Pare enhances the survival rate of rats with pulmonary I/R injury

**Figure 7:**
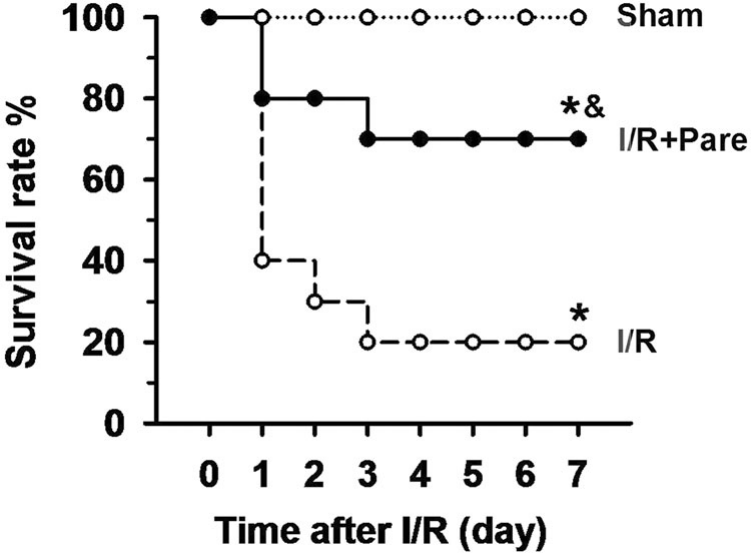
Assessment of the rat survival rate after pulmonary I/R injury with/without Pare pretreatment. Thirty rats were evenly divided into three groups with 10 rats in each group: the Sham group, the I/R only group, and the I/R + Pare group. The number of surviving rats over a 7‐day period after pulmonary I/R was counted and the survival rate was calculated using the Kaplan–Meier method with a logrank test. 0 day means the time point at 2 h after reperfusion on the day of surgery. 80% means 8 out of 10 rats survived, 40% means 4 out of 10 rats survived, and so on. ^*^ *p* < .05, compared to the sham group; ^&^ *p* < .05, compared to the I/R group. I/R, ischemia/reperfusion; Pare, Parecoxib.

Finally, the effectiveness of Pare in improving the survival rate of rats with I/R injury was investigated. The results revealed that 20% of the rats in the I/R group survived beyond 7 days; however, Pare markedly increased the post‐I/R survival rate to ∼68% (Figure 7, *n* = 10, *p* < .05).

## 4 DISCUSSION

Pulmonary I/R injury is involved in a number of clinical conditions, such as lung transplantation, acute massive pulmonary embolism following thrombolytic therapy, the re‐expansion of chronically collapsed lungs from pneumothorax, or pleural effusion and cardiopulmonary bypass.^23^ Due to the high mortality rate, the identification of strategies with which to prevent and treat I/R‐induced lung injury is of utmost clinical significance. The present study focused on investigating the potential roles of Pare in pulmonary I/R injury. The results revealed that the pulmonary ventilation function in the Pare‐pretreated group was markedly improved relative to that in the I/R group. In addition, the histological damage was much less prominent with Pare pretreatment than in the rats with I/R not treated with Pare. Additionally, Pare attenuated I/R‐induced oxidative stress and inflammatory responses and improved the survival rate of rats with I/R injury. Therefore, the application of Pare before ischemia appears to protect rats against pulmonary I/R injury, possibly through the suppression of the ERK/NF‐κB pathway and further activation of the HIF‐1α pathway.

The excessive generation of free radicals is involved in various I/R injuries. MDA is a breakdown product of unsaturated fatty acid lipid‐peroxidation by oxygen free radicals. MDA is often used as a key indicator to evaluate the generation of oxygen free radicals or the degree of organ/tissue damage.^24^ SOD, a critical oxygen free radical scavenger, is directly related to the antioxidant capacity of organisms.^25^ CAT is an antioxidant enzyme that plays a key role in the defense against oxidative stress via the detoxification of hydrogen peroxide. Polyethylene glycol‐CAT has been shown to protect against oxidative stress‐induced cytotoxicity, sustain cell metabolism and alleviate pulmonary I/R damage.^26^ GSH‐Px is another key cellular antioxidant enzyme that protects against oxidative stress.^27^ The present study found that, compared to the control group, the pulmonary MDA level was markedly induced, and the activities of pulmonary SOD, CAT, and GSH‐Px were also markedly reduced in the two I/R groups. However, compared to the I/R only group, the pulmonary MDA level was markedly reduced, and the activities of pulmonary SOD, CAT, and GSH‐Px were notably enhanced in the Pare‐pretreated I/R group. The aforementioned findings demonstrate that oxidative stress is extensively involved in rats suffering from I/R, and Pare pretreatment decreases oxidative stress, thus alleviating I/R‐induced injury.

Mitochondrial dysfunction has been implicated in the pathological progression of I/R‐induced organ damage, presenting as the disruption of mitochondrial homeostasis and the depolarization of MMP.^20^ Moreover, the mitochondria are the major source of ROS, which are normally regarded as the harmful by‐product of aerobic metabolism.^28^ High levels of ROS have been shown to be induced in an ischemic environment and further lead to tissue damage.^20^ Therefore, investigating the intracellular ROS level and MMP change is crucial for evaluating the protective role of Pare in pulmonary I/R injury. In the present study, I/R significantly increased the intracellular ROS level and the number of depolarized cells, indicating a decrease in MMP and a disorder of mitochondrial function. Notably, Pare pretreatment effectively decreased the ROS level and elevated MMP, exhibiting a potent protective role in mitochondrial function.

Abundant inflammatory responses also play significant roles during the pathological process of I/R. Various types of cells are involved in the I/R process, causing the infiltration of inflammatory cells, as well as the production of proinflammatory mediators, including TNF‐α, IL‐6, and IL‐8. TNF‐α, a key initiating factor in pulmonary I/R injury, exerts harmful effects on lung vascular permeability.^29^ IL‐6, a pleiotropic cytokine that mediates a range of functions, including inflammatory responses, can be promptly and instantaneously generated upon infection or injury, resulting in the host defense through the activation of acute‐phase responses, hematopoiesis, and immunoreactivities.^30^ The knockdown of IL‐6 mitigates the severity of acute lung injury and the degree of edema caused by intestinal I/R in rats.^31^ IL‐8 is a major chemotactic and activating cytokine for neutrophils in the lungs. It has been found that a neutralizing monoclonal antibody against IL‐8 prevents the infiltration of neutrophils and lung damage.^32^ In addition, the release of IL‐8 during lung I/R is closely related to the graft function at the early stages following lung transplantation.^33^ The overproduction of TNF‐α, IL‐6, or IL‐8 can promote the aggregation of neutrophils, increase the permeability of vessels, and cause interstitial edema of the lungs, which further disrupts the gaseous exchange and exacerbates lung damage. In the present study, the TNF‐α, IL‐6, and IL‐8 contents in the serum in the two I/R groups were notably higher than those in the control. However, compared to the I/R group, the TNF‐α, IL‐6, and IL‐8 contents in the Pare‐pretreated group were markedly decreased. The IHC staining of TNF‐α further verified this conclusion. On the whole, these results indicate that the Pare‐mediated alleviation of I/R injury may be associated with its inhibitory effects on inflammation.

I/R activates multiple signal‐transduction pathways, such as ERK and NF‐κB. ERK is a pivotal mediator during signal transduction in response to various tissue injuries and cellular stresses.^34^ A recent study revealed activated EKR and the induction of TNF‐α in lung I/R injury.^23^ In addition, the expression of proinflammatory cytokines in microvessels was previously shown to be induced following cerebral ischemia by activating ERK signaling, while the inhibition of the ERK‐involved mechanism markedly decreased the infarct size.^35^ Phosphorylated ERK immunostaining was principally detected in renal tubular cells accompanied by significant renal insufficiency, histopathological alteration, oxidative stress, and the formation of mitochondrial complexes following renal I/R injury, indicating an activation of the ERK pathway.^36^ It has been found that ERK activation can further facilitate the disintegration of NF‐κB/IκB complexes, inducing the activation of the NF‐κB pathway.^37^

NF‐κB is an important and ubiquitous transcription factor involved in the regulation of the expression of numerous cytokines in lung injury.^38^ Upon activation by inflammatory stimuli, phosphorylated IκB disassociates with cytoplasmic NF‐κB, and enables its migration to the nuclei. The intranuclear NF‐κB then directly assembles on enhancer elements of target genes and induces the transcription of proinflammatory genes.^38^ A recent study demonstrated that NF‐κB signaling and a number of proinflammatory factors were induced during lung I/R, as I/R induced IKK‐β phosphorylation, decreased IκB expression and activated NF‐κB signaling.^23^ In addition, a previous in vitro model using rat pulmonary artery endothelial cells, mimicking the in vivo lung I/R model, also demonstrated that hypoxia‐reoxygenation promoted the phosphorylation of ERK1/2 and the trans‐activation of NF‐κB.^39^ In the present study, pulmonary I/R markedly enhanced the activation of ERK and NF‐κB by several folds when compared to the sham group. However, Pare preadministration significantly attenuated the I/R‐induced activation of ERK and NF‐κB, indicating that the protective effects of Pare against I/R injury may be mediated via the inhibition of ERK/NF‐κB signaling.

HIF‐1α is known as a pivotal transcriptional factor in response to oxygen level changes and it activates several genes that facilitate the adaptation to ischemia and oxidative stress.^40^ The activation of HIF‐1α promotes cell survival under hypoxic conditions involving angiogenesis, cell respiration, and glucose metabolism.^41^ The protective effects of HIF‐1α have been indicated in various organs damaged by I/R. A previous study using a rat model of cerebral I/R injury demonstrated that the augmentation of HIF‐1α expression markedly attenuated brain edema and improved the integrity of the brain‐blood barrier.^42^ Dexmedetomidine treatment has been shown to effectively increase the HIF‐1α level and alleviate I/R‐induced injury.^43^ In the present study, I/R significantly induced HIF‐1α expression, while Pare pretreatment further amplified this induction, indicating the activation of HIF‐1α signaling in the Pare‐mediated protective effects against lung I/R injury.

Pare, as a selective and potent inhibitor of COX‐2, has a wide range of applications for postoperative pain relief in clinical practice.^44^ However, the prophylactic use of Pare has been reported in clinical studies.^45^, ^46^ A possible mechanism underlying the role of Pare in I/R injury may lie in its inhibitory effects on COX‐2 and prostaglandin synthesis.^47^ A number of studies have demonstrated the protective effects of Pare on various tissues in animal models of I/R injury.^11^, ^12^, ^13^, ^14^ Such a protective role of Pare is mainly mediated through anti‐inflammation, antioxidation, as well as the regulation of apoptosis and autophagy,^11^, ^12^, ^13^, ^14^ highlighting the superiority and potential values of Pare in clinical application. However, the potential role of Pare in pulmonary I/R injury and the underlying mechanisms have rarely been reported. The present study provides insight into this matter, demonstrating that Pare significantly improved lung ventilation function, attenuated oxidative stress, decreased the inflammatory responses, protected the lungs from I/R‐induced tissue damage, and improved the rat survival rate, possibly via the suppression of ERK/NF‐κB and the activation of the HIF‐1α pathway.

In conclusion, the present study demonstrated that Pare effectively improved the pulmonary ventilation function, attenuated oxidative stress, protected mitochondrial function, decreased inflammatory responses, attenuated histopathological damage, and enhanced the rat survival rate, possibly via the suppression of ERK/NF‐κB and activation of HIF‐1α signaling, indicating a potential therapeutic effect of Pare on lung I/R injury. Results from the current study are consistent with an earlier published work.^48^ Particularly, we have extensively expanded the research scope and confirmed for the first time the beneficial role of Pare against pulmonary I/R at the mitochondrial, histopathological, and animal levels. Additionally, we have first demonstrated the involvement of ERK/NF‐κB/HIF‐1α pathways that are implicated in tissue injury. However, this study still has certain limitations. For example, what is the underlying mechanism mediating the inductive effect of Pare on HIF‐1α expression? Would this inductive effect on HIF‐1α be also applied to humans? Would preadministration of Pare produce effective and efficient protection against IR injury during lung transplantation? Further research are urgently needed to answer these questions.

## AUTHOR CONTRIBUTIONS

Jiantao Guo and Yiping Yang designed the study, performed the experiments, analyzed the data, wrote the manuscript, and approved the final submission.

## CONFLICT OF INTEREST

The authors declare no conflict of interest.

## Data Availability

Data are available upon reasonable request.
